# Effects of weather and moon phases on emergency medical use after fall injury: A population-based nationwide study

**DOI:** 10.1371/journal.pone.0261071

**Published:** 2021-12-31

**Authors:** Min Ah Yuh, Kisung Kim, Seon Hee Woo, Sikyoung Jeong, Juseok Oh, Jinwoo Kim, Sungyoup Hong

**Affiliations:** 1 Department of Emergency Medicine, Daejeon St Mary’s Hospital, The Catholic University of Korea College of Medicine, Seoul, Republic of Korea; 2 BioBrain Inc, Daejeon, Republic of Korea; 3 Department of Emergency Medicine, Incheon St Mary’s Hospital, The Catholic University of Korea College of Medicine, Seoul, Republic of Korea; 4 Department of Emergency Medicine, Uijeongbu St Mary’s Hospital, The Catholic University of Korea College of Medicine, Seoul, Republic of Korea; 5 Department of Emergency Medical Service, Daejeon Health Institute of Technology, Daejeon, Republic of Korea; Tsinghua University, CHINA

## Abstract

**Background:**

Previous studies reported that changes in weather and phases of moon are associated with medical emergencies and injuries. However, such studies were limited to hospital or community level without explaining the combined effects of weather and moon phases. We investigated whether changes in weather and moon phases affected emergency department (ED) visits due to fall injuries (FIs) based on nationwide emergency patient registry data.

**Methods:**

Nationwide daily data of ED visits after FI were collected from 11 provinces (7 metropolitan cities and 4 rural provinces) in Korea between January 2014 and December 2018. The daily number of FIs was standardized into FI per million population (FPP) in each province. A multivariate regression analysis was conducted to elucidate the relationship between weather factors and moon phases with respect to daily FPP in each province. The correlation between weather factors and FI severity was also analyzed.

**Results:**

The study analyzed 666,912 patients (418,135 in metropolitan and 248,777 in rural areas) who visited EDs on weekdays. No regional difference was found in age or gender distribution between the two areas. Precipitation, minimum temperature and wind speed showed a significant association with FI in metropolitan areas. In addition, sunshine duration was also substantial risk factors for FI in rural areas. The incidence of FIs was increased on full moon days than on other days in rural areas. Injury severity was associated with weather factors such as minimum temperature, wind speed, and cloud cover.

**Conclusion:**

Weather changes such as precipitation, minimum temperature, and wind speed are associated with FI in metropolitan and rural areas. In addition, sunshine duration and full moon are significantly associated with FI incidence only in rural areas. Weather factors are associated with FI severity.

## Introduction

Accurate prediction of the need for emergency medical care is critical to provide appropriate services for patients with injuries. Therefore, many countries have an emergency medical system data registry collected during pre-hospital and in-hospital phases to design emergency medical service (EMS) and implement public health monitoring and planning.

Fall injury (FI) is the second major cause of accidental or unintended injury-related deaths worldwide [1]. The mechanism of injury for falls is vertical deceleration due to the force of gravity high place or loss of balance on a slippery surface. FIs in older or disabled individuals increase in winter due to low temperatures and long nights [2]. The slippery ground caused by melting ice, snow-covered ice, and ice is a typical cause of FI in winter [3]. The incidence of FIs is known to have a seasonal variation depending on geographical location, such as countries with a cold climate (Russia, Canada, Sweden, Finland, and Norway) [4, 5] and countries with a warm tropical climate, such as Hong Kong [6].

Weather conditions have been reported to influence the occurrence of trauma and disease. Poor weather conditions may lead to traumatic events [7, 8]. However, other studies reported that outdoor activities even in good weather are related to increased incidence of all kinds of injuries [9]. If we target FI only, snowfall and icy surfaces were associated with FIs in late autumn and winter [6, 7, 10]. But another study reported the increased frequency of FIs was found in better weather with medium mean air temperature and atmospheric pressure during warm season [4].

A full moon has been reportedly associated with potential emergency department (ED) visits after traffic accidents [11] and mortality after motorcycle crashes and accidents [12]. However, Stomp et al. reported that phases other than full moon increased ED visits after all kinds of trauma [9]. Such difference might be attributed to the use of nationwide statistics of road car accidents in two of the three studies, whereas Stomp et al. [9] used all types of trauma data from one ED located in a small suburban area of Netherlands. Therefore, findings from these studies were limited by small sample size in specific regions, restricted data sources or target injury.

In summary, previous studies evaluated the role of weather and lunar phases; however, the findings were limited to specific region or involved a small sample size. To prevent unwanted medical errors due to ED crowding and provide prompt and appropriate EMS, it is vital to foresee the demand for emergency medical use due to FIs. The primary purpose of this study was to assess the FI prevalence according to regional characteristics, weather changes and moon phases using the nationwide longitude data. The secondary goal was to determine the effects of weather factors and moon phases on FI severity.

## Materials and methods

### Study design and data collection

To analyze the association of FI incidence with weather factors and lunar phases, a nationwide epidemiological analysis was conducted based on emergency department usage data obtained from all emergency centers in Korea. The population of mainland Korea and its affiliated islands of 99,000 km^2^ is approximately 50 million. Korea has a total of 420 registered ERs that are open to all beneficiaries without restriction. The National Emergency Department Information System (NEDIS) operated by the National Emergency Medical Center (NEMC) prospectively collected data of patients who visited all Korean EDs since 2005. This study used the NEDIS data of patients who visited emergency centers after FI, including age, gender, region of occurrence, onset time, injury mechanism, injury severity with Korean Triage and Acuity Scale (KTAS), and outcome of emergency care from January 2014 to December 2018. No personal identifier was included in these data. Data were stored in a secured personal computer.

KTAS score consists of five levels of acuity: level 1 (resuscitation), level 2 (urgent), level 3 (emergent), level 4 (non-urgent), and level 5 (delayed). The KTAS was developed as a single triage tool for emergency patients in Korea and has since become nationalized [13].

Daily weather data including precipitation, minimum temperature, mean wind speed (wind speed), sunshine and fog duration, and cloud cover were obtained from the Korea Meteorological Agency (KMA). Daily precipitation was calculated as the sum of hourly measurements for 24 hours. Cloud cover was calculated in integers ranging from 0 to 10 tenths based on visual cloud cover observations from each observation site. A weather station located in the capital city of each province in Korea was selected to represent the weather data collection point. Moon phase data were obtained from a website (https://www.timeanddate.com/moon/phases/south-korea/).

First, pediatric patients under the age of 15 years were excluded from the collected data. FI patients on weekdays excluding Saturday, Sunday, and public holidays (New Year’s Day, Lunar New Year’s Day, Children’s Day, Korean Independence Day, Lunar Thanksgiving Day and Christmas) were also excluded from this study. We analyzed data from a total of 743 nights (182 new moon days, 186 first quarter and full moon days, and 189 3rd quarter nights). The full moon period was determined for three days starting from one night before to one night after the peak full moon night. The same rule was applied to other moon phases.

Annual mid-population data of each province were obtained from the central organization for Statistics of Korea (http://kostat.go.kr/portal/eng/index.action).

### Outcome measurement

We counted the daily number of patients who visited EDs after a FI for each province. The number of daily FIs was standardized into FIs per million population (FPP) by dividing with an annual mid-population of the province. This study compared FPP and severity of FI between two regions: 1) metropolitan areas consisting of provinces with a population of more than one million; and 2) rural areas without metropolitan cities within the perimeter of the provincial limit (S1 Fig). The primary outcome was the daily number of ED visits due to FI. It was calculated as the number of patients per million people. The secondary outcome was injury severity of patients and was determined by the mean KTAS score of all daily FI patients in the designated area.

### Statistical analysis

The chi-square test is extremely sensitive to sample size. If the sample size is too large (> 500), any small differences appear statistically significant [14]. Hence, we used Cramer’s V statistics instead of Chi-square test to estimate the association of ordinary factors between two regions. The means of the continuous variable were compared using Student’s t-test between two regions. The number of FI events in a fixed time interval was modeled using Poisson distribution, and thus a generalized linear model (GLM) with a Poisson distribution and log-linear function was used to assess the significance of association between dependent variable (FPP for each day) and independent variables including weather factors and lunar phases. All variables with a *p* value < 0.2 in the univariate analysis were entered into multivariate analysis. The incidence risk ratios (IRRs) and their 95% confidence intervals (CIs) were calculated for each independent variable. Theoretical FI incidence-factor curve was approximated via nonlinear curve fitting with Boltzmann sigmoidal function and illustrated with scatter plots. The correlation between weather factors and daily mean KTAS was analyzed using Pearson correlation coefficients. All statistical analyses were analyzed using Origin Pro (OriginLab, Northampton, MA) and Rstudio 1.4.1717 (RStudio Inc, Boston, MA). Statistically significant difference was indicated by a *p* value 0.050 or less.

### Ethical approval

The study protocol was reviewed and approved by the Institutional Review Board of Daejeon St Mary’s Hospital, The Catholic University of Korea (DC21ZIS10034).

## Results

### Demographic characteristics of the study subjects

**Fig 1:**
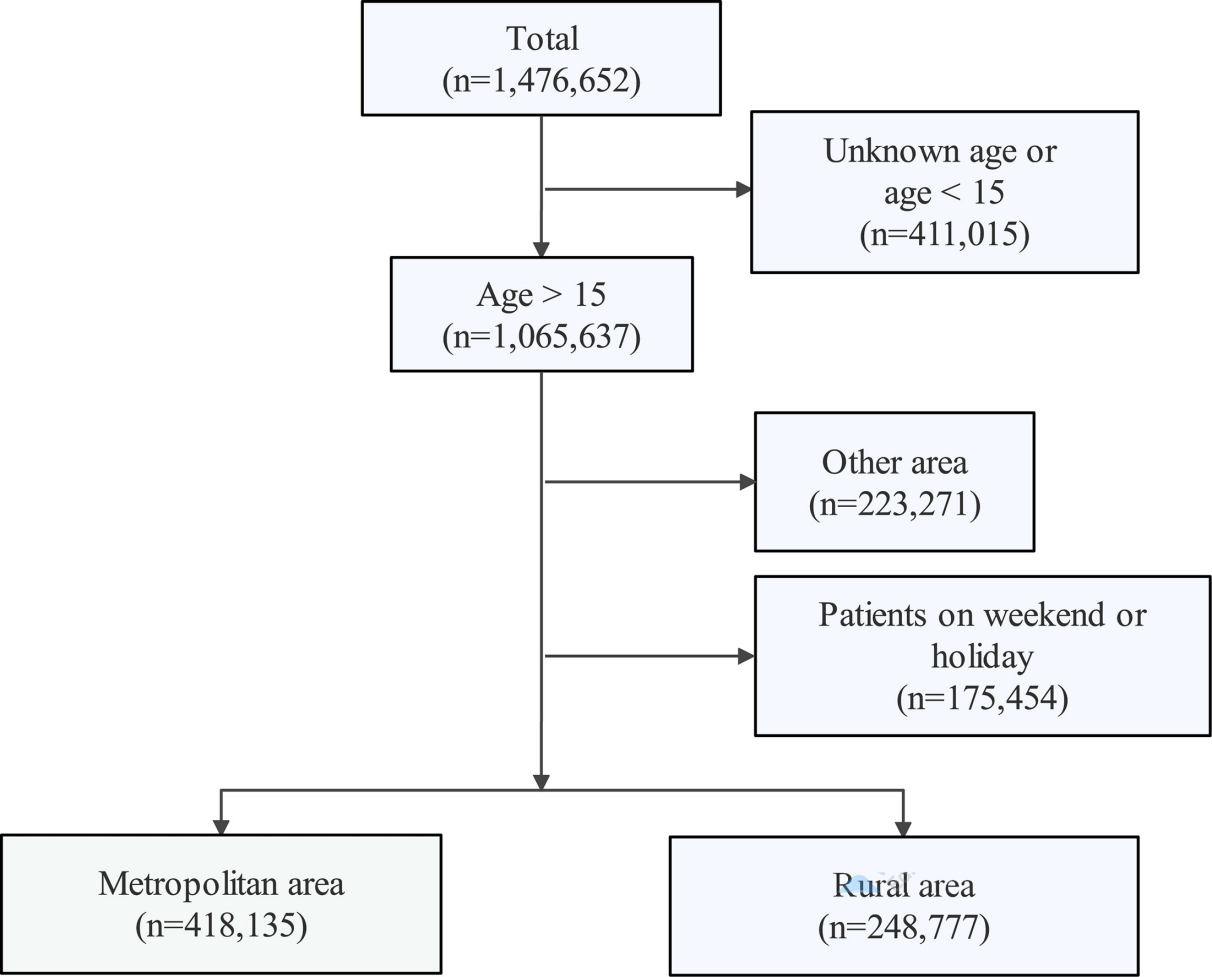
Schematic diagram showing the selection of study population for this study.

Of 1,476,652 people included in the registry with FI, 1,065,637 were older than 15 years in Korea between Jan 2014 and Dec 2018 (Fig 1). FPPs were significant higher on weekends than on weekdays (P < 0.010, S2 Fig). Hence, we excluded FI cases on weekend and holidays to prevent bias. Finally, 666,912 patients (418,135 in metropolitan and 248,777 in rural areas) on weekdays were analyzed in this study. The mean age of patients finally enrolled was 54.4 ± 20.4 years. There was no significant association with age distribution between the two regions, but the proportion of male patients was significantly higher in the rural areas (Table 1, *P* < 0.01). The distribution of FI between the two regions was balanced with no monthly difference. Of the total patients, 69, 563, 66, 755, 69, 573, and 69,783 patients suffered FIs on the new moon, 1^st^ quarter, full moon, and 3rd quarter days, respectively. A notably higher number of FIs occurred on full moon days (Table 1, *P* < 0.010) and a significantly higher proportion of patients were brought to ED in an ambulance in the rural areas (Table 1, *p* = 0.017).

**Table 1:** Demographic features of subjects who visited ED after a fall injury.

Variable		Metropolitan	Rural	Cramer’s V or *p* value
		(n = 418,135)	(n = 248,777)	
		N (%)	N (%)	
Age	15–19	25,423 (5.0)	7,299 (5.7)	V = 0.001
	20–24	28,389 (5.6)	7,864 (5.5)	
	25–29	29,192 (5.6)	7,795 (4.7)	
	30–34	29,566 (5.8)	7,988 (5.0)	
	35–39	27,869 (5.5)	7,780 (5.6)	
	40–44	30,840 (6.1)	8,706 (6.5)	
	45–49	36,172 (7.1)	10,245 (7.7)	
	50–54	43,583 (8.6)	12,272 (9.1)	
	55–59	48,404 (9.5)	13,521 (9.1)	
	60–64	38,986 (7.7)	10,742 (7.3)	
	65–69	35,154 (6.9)	9,593 (6.3)	
	70–74	37,268 (7.3)	10,214 (6.8)	
	75–79	39,169 (7.7)	10,960 (7.9)	
	80–84	31,347 (6.2)	8,844 (6.6)	
	85–89	17,844 (3.5)	5,034 (3.7)	
	90–94	6,660 (1.3)	1,893 (1.4)	
	95–99	1,393 (0.3)	409 (0.3)	
	100–104	183 (0.0)	59 (0.1)	
	105–109	24 (0.0)	13 (0.0)	
	110–120	8 (0.0)	3 (0.0)	
Sex	Male	258,579 (51.0)	132,101(53.1)	P< 0.010
Month	Jan	44,515(8.8)	12,288 (8.7)	V = 0.020
	Feb	38,357 (7.7)	10,381 (7.4)	
	Mar	39,581 (7.8)	10,448 (7.4)	
	Apr	40,136 (7.9)	10,939 (7.8)	
	May	44,224 (8.7)	12,420 (8.8)	
	Jun	38,029 (7.5)	10,928 (7.7)	
	Jul	39,968 (7.9)	11,315 (8.0)	
	Aug	41,564 (8.2)	12,131(8.6)	
	Sep	43,425 (8.6)	12,371 (8.8)	
	Oct	46,051 (9.1)	13,404 (9.5)	
	Nov	42,183 (8.3)	11,675 (8.3)	
	Dec	49,410 (9.7)	12,931 (9.2)	
Moon phase	New moon	44,461 (10.6)	25,102 (10.1)	*p* <0.010
	1^st^ quarter	41,531 (9.9)	25,224 (10.1)	
	Full moon	43,107 (10.3)	26,466 (10.6)	
	Last quarter	44,059 (10.5)	25,679 (10.3)	
	other	244,713 (58.6)	146,306 (58.8)	
Route	Ambulance	439,439 (86.6)	124,180 (87.9)	*p* = 0.017
	Private car	65,574 (12.9)	16,504 (11.7)	
	Ambulation	2,227 (0.4)	520 (0.3)	
KTAS	1	920 (0.80)	223 (0.67)	V = 0.076
	2	5334 (4.61)	1195 (3.59)	
	3	34519 (29.83)	12620 (37.95)	
	4	63769 (55.11)	15897(47.81)	
	5	11155 (9.64)	3317(9.97)	
Mental	Alert	487775 (96.1)	137035 (97.0)	V = 0.022
	Verbal response	11928 (2.4)	2358 (1.7)	
	Pain response	5038 (1.0)	1089 (0.8)	
	Unresponsive	2544 (0.5)	738 (0.5)	
SBP		134.0 ± 26.6	135.7 ± 27.1	*p* = 0.001
DBP		79.8 ± 19.3	80.7 ± 19.5	*p* <0.010
PR		88.8 ± 40.4	89.5 ± 19.5	*p* <0.010

KTAS, Korean triage and acuity scale; SBP, systolic blood pressure; DBP, diastolic blood pressure; PR, pulse rate.

In the rural areas of this study, the proportion of severe patients with KTAS scores of 1 to 2 was significantly lower, and the proportion of patients with KTAS scores from 3 to 5 was higher (Table 1, Cramer’s V = 0.076). The proportion of mentally alert patients was higher (V = 0.022) than in the metropolitan areas. The systolic and diastolic blood pressure and pulse rate per minute of FI patients in the rural areas were significantly higher in rural areas than in metropolitan areas.

### Relationship of FI incidence with weather and moon phase

**Fig 2:**
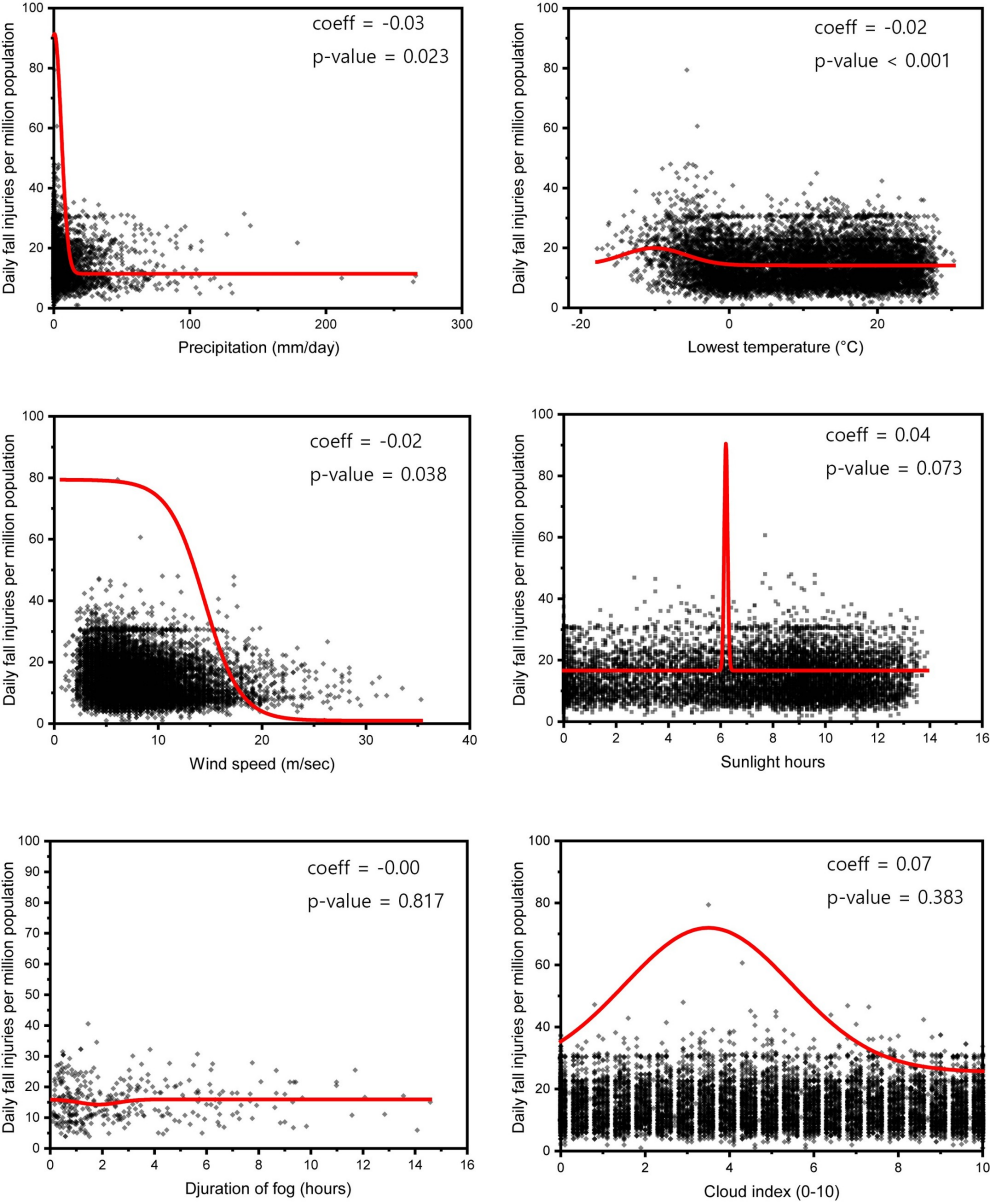
Scatter plots of the number of ED visits per million people after fall injuries versus weather components in metropolitan areas of Korea. A theoretical Gaussian regression line estimated by nonlinear curve fitting with the Boltzmann sigmoidal function is shown in red.

**Fig 3:**
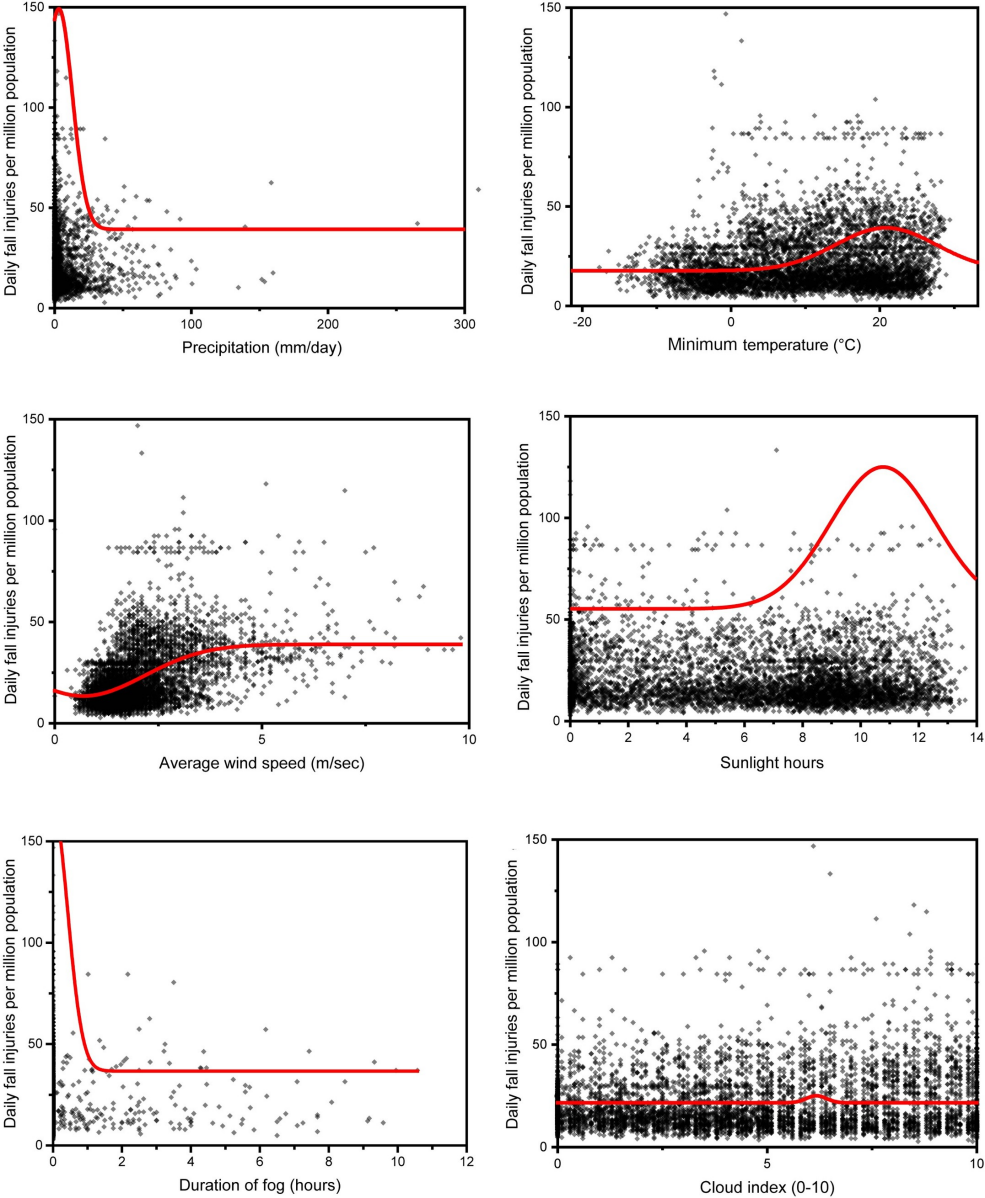
Scatter plots of the number of ED visits per million people after fall injuries versus weather components in rural areas of Korea. A theoretical Gaussian regression line estimated by nonlinear curve fitting with the Boltzmann sigmoidal function is shown in red.

Pooled associations of the daily FPP with weather and moon phase are presented in Table 2, Fig 2 (metropolitan area), and Table 3, Fig 3 (rural area). Among weather factors, precipitation, minimum temperature, and wind speed showed a significant association with FI in metropolitan areas (Table 2). FIs occurred frequently on days with lower precipitation, lower minimum temperature, and low-wind days in metropolitan areas (Fig 2). In rural areas, FIs have been shown to increase significantly on days with lower precipitation levels, higher minimum temperatures, higher wind speed and longer sunshine duration (Table 3, Fig 3).

**Table 2:** Multivariate regression analysis of relationships between weather factors and moon phase with fall injuries in metropolitan areas.

	Univariate analysis		Multivariate analysis		
	IRR	*p* value	IRR	*p* value	CI
Precipitation	0.99	0.043	0.93	0.045	0.93–0.97
Minimum temperature	0.98	<0.001	1.27	0.005	0.97–0.98
Wind speed	0.78	0.003	0.80	<0.001	0.95–1.01
Cloud cover	1.07	0.269			
Sunshine duration	1.03	0.020	1.01	0.516	1.01–1.02
Fog duration	1.00	0.677			
Moon phase (versus full moon)					
New moon	1.10	0.567			
1st quarter	1.12	0.532			
3rd quarter	1.25	0.724			
Interaction effects					
precipitation:minimum			1.00	0.001	1.00–1.01
precipitation:wind			1.01	0.755	1.01–1.01
precipitation:sunshine			1.01	0.414	1.01–1.01
minimum:wind			0.99	0.001	0.99–1.00
minimum:sunshine			1.02	0.023	1.01–1.03
wind:sunshine			0.99	0.131	0.99–0.99

IRR, incidence risk ratio; SE, standard error; CI, confidence interval.

**Table 3:** Multivariate regression analysis of relationships between weather factors and moon phase with fall injuries in rural areas.

	Univariate analysis		Multivariate analysis		
	IRR	*p* value	IRR	*p* value	CI
Precipitation	0.96	<0.001	0.98	<0.001	0.90–0.97
Minimum temperature	1.25	<0.001	1.20	<0.001	0.99–1.51
Wind speed	1.24	<0.001	1.18	<0.001	1.23–1.25
Cloud cover	2.01	0.383			
Sunshine duration	1.19	<0.001	1.20	0.040	0.67–2.20
Fog duration	0.03	0.817			
Moon phase (versus full moon)					
new moon	0.75	0.002	0.78	0.043	0.61–0.85
1st quarter	0.61	0.948	0.60	0.556	0.95–1.02
3rd quarter	0.68	0.984	0.68	0.984	0.99–1.03
Interaction effects					
precipitation:wind			1.01	<0.001	1.01–1.01
precipitation:minimum			1.00	<0.001	1.00–1.00
precipitation:sunshine			0.98	<0.001	0.96–0.99
minimum:wind			1.01	<0.001	1.01–1.02
minimum:sunshine			1.00	0.590	1.00–1.00
wind:sunshine			1.08	<0.001	1.06–1.11
new moon:precipitation			1.01	0.027	0.00–14.6
new moon:wind			1.02	0.028	0.79–1.16
new moon:sunshine			0.99	0.055	0.02–3.13

IRR, incidence risk ratio; SE, standard error; CI, confidence interval.

The distribution of FI patients was compared according to moon phase. The frequency of FIs was higher on full moon days than on new moon days in rural areas (Table 1, p < 0.010). Full moon was a significant predictor of FIs in univariate analysis in rural area (Table 3, *p* = 0.048) but not significant in multivariate analysis. Based on the interaction analysis, the new moon phase showed a significant interaction with precipitation and wind speed in rural areas. However, there was no significant difference in the incidence of FI as similar FIs occurred on all days in metropolitan areas (Tables 1 and 2).

### Correlation of weather factors with FI severity

The severity of FI was measured using KTAS assessed upon ED arrival. KTAS 1 refers to a state warranting emergency resuscitation, and KTAs 5 indicates absence of emergency. Injury severity (daily mean KTAS for each province) was significantly correlated with minimum temperature and wind speed and thus the injury severity was increased on cold windless days in both areas (Table 4). Additionally, in rural areas, the daily mean KTAS was significantly correlated with cloud cover. Precipitation, sunshine, and fog duration were not associated with the severity of FI in the rural or metropolitan areas. There was no significant difference in mean KTAS depending on the lunar phase in the metropolitan or rural areas (*p* = 0.394, *p* = 0.457, respectively).

**Table 4:** Results of Pearson’s correlation analysis between injury severity (mean KTAS) with weather factors.

		Precipitation	Minimum temperature	Mean wind speed	Cloud cover	Sunshine duration	Fog duration
Metro	CC	0.006	0.049	0.157	-0.045	-0.012	<0.001
	*p* value	0.701	0.003	<0.001	0.700	0.957	0.995
Rural	CC	-0.008	0.065	0.108	0.298	0.005	0.030
	*p* value	0.718	0.004	<0.001	0.007	0.809	0.175

CC, correlation coefficient.

## Discussion

During the study period of five years, we found that the prevalence and severity of FI were associated with multiple weather factors such as daily precipitation, minimum temperature, and wind speed in both metropolitan and rural areas. However, we found that the longer the sunshine duration was linked with the higher FI in rural area. Moon phases were weakly associated with FI, especially in rural areas. The FI severity was closely related to weather factors.

This study enrolled the largest dataset ever collected to determine the association between weather factors and ED visits due to FI in all 11 provinces of Korea over a 5-year period. A previous study conducted in a small city of 23,000 people in northern Netherlands reported that better weather conditions were associated with the incidence of all types of trauma [9]. The study location was similar to rural South Korea, where weather components including maximum temperature, sunshine duration, humidity, and precipitation were associated with all kinds of injury. The present study also found that precipitation, minimum temperature, and wind speed were typically related to FI. Additionally, sunshine duration was a significant predictor of FI in rural areas with high agricultural activities. These findings suggest that it is essential to consider a variety of factors such as geographic location, main industry in the region, and weather changes during the investigation of injury prevalence.

Ramgopal et al. [8] investigated the association of weather factors with all EMS dispatches using longitudinal data of ambulance transport in western Pennsylvania and reported increased EMS responses with rising temperature, snowfall, and rain based on a stratified analysis of seasonal variables and a day-of-the-week effect week. We found that additional factors such as wind speed, cloud cover, and sunshine duration were associated with emergency resource use after FI. However, seasonal changes were not included as independent variables in this study because changes in minimum temperature and precipitation implicated seasonal variations in weather. We also excluded FI on weekends and holidays because of increased trauma due to enhanced outdoor leisure activity and distant travel on days that might act as a confounding variable. We expected no major challenges in the analysis of FIs on weekdays because of a sufficient number of cases using 5-year large-scale longitudinal data for at the nationwide level.

Stomp et al. [9] reported that better weather conditions in rural areas were associated with the incidence of all traumas. Our analysis also found that the frequency of FIs in rural areas was increased under less precipitation, higher minimum temperature, and longer sunshine duration such as busy farming seasons.

This is the first study to compare FI-related factors between developed metropolitan cities and rural areas. During the course of our study, another research paper reported the correlation between weather changes and FI in a small Russian city [4]. It was the only longitudinal study for FIs like this study but was limited by geographic location of the study area or by small number of subjects. The daily average of outdoor falls in the cold season was 20.2 per 100,000 people and the slippery surfaces covered with wet snow or ice and temperatures between -7.0°C and -0.7°C were risk factors. As mentioned above, our study results showed a distinct increase in FIs according to regional characteristics, with a lower temperature triggering falls on slippery surface in metropolitan areas, and a higher temperature during increased agricultural activity in rural areas associated with increased FIs. They also reported that the FIs were increased when the 12-hour precipitation was greater than 0.4 mm; however, the present study showed that the FIs were increased under low precipitation. This difference is probably explained by the falling of snow leading to slippery surfaces in Russia with a high altitude, whereas in Korea located in mid-latitude weather, rain accompanied by summer storms with strong winds reduced the frequency of outdoor activities. Northern Russia is located at the highest latitude among countries in the world. As the highest temperature in summer was near zero, the study failed to reflect changing weather patterns in mid-latitude areas with four clear seasons.

Good weather conditions accompanied by active agricultural activities and increased night visibility under moonlight on a full moon might be associated with FIs in rural areas. A moon phase occurs every 29.53 days and 12.37 times in a year. The four principal moon phases include: new moon, the 1st quarter, full moon, and the last quarter. Moon phases are known to drive periodic changes in nighttime illumination, geomagnetic fields, gravitational pull, and other factors associated with major meteorological and biological changes [15]. We found that FI-related ED visits on full moon days were significantly increased than on new moon days only in rural areas. Two of the four rural provinces in this study border the sea and another province is an island with active fishing activities (S1 Fig). The full moon is a time of full tide and active fishing activities due to vertical migration of fishes [16]. Therefore, active nighttime activities and fishing activities might increase FIs in rural areas. However, FIs in metropolitan areas were less affected by lunar phase due to good visibility under night light that offset the effects of lunar phases.

A previous study from Japan revealed a significant increase in the risk of emergency transport after traffic accidents on full moon days among those aged ≥40 years [11]. This finding is consistent with the results of our study showing a significant increase in FIs during full moon days especially in rural areas where the elderly individuals reside under weak artificial lighting at night. A population-based double control study conducted in the United States reported that deaths from motor traffic accidents are more frequent on full moon nights [12]. The authors postulated that a full moon might be associated with speeding, long distances, and unknown routes, resulting in more frequent deaths. In our study, FIs were increased on full moon days only in rural areas with weak artificial lighting, suggesting that increased visibility and outdoor activity under moonlight on full moon days are associated with increased FIs. The analysis of interaction between lunar phases and weather factors showed that the new moon phase interacted with precipitation, wind speed, and cloud cover, which is consistent with the findings of a previous study showing an increased number of storms during new moon phases [17]. The finding suggests that the decrease in FIs in rural areas during new phases may be a result of weather changes. Thus, the effect of the lunar phase is complex with increased near-field vision due to moonlight mainly in rural areas and secondary weather changes associated with lunar phases.

We found that weather factors were correlated with FI severity measured by KTAS, a unified triage scoring system. KTAS is a five-level triage scale developed in Korea based on Canadian Triage and Acuity Scale (CTAS) and the score is a strong predictor of severity of patients with higher 30-day mortality [18].

The strength of the study is that it is a population-based analysis of longitudinal data involving FIs in a mid-latitude country with four clearly distinguished seasons. A few unknown environmental factors may confound the study results. Future studies should use more complex modeling methods and evaluate the effects of moon phases and weather changes. Patients sustaining FIs may visit the ED the next day or later instead of on the day of injuries. Morency et al. [19] reported a significant increase in outdoor falls on days 1–3 after falling temperatures or snowfall. Therefore, it might be a challenge to compare changes in weather phenomena and patients visiting the hospital on the same day. We believed that the interval between the weather change and FIs is not a hindrance because of the gradual changes in weather and FI incidence over a period of several days. We enrolled subjects regardless of indoor or outdoor injuries because exposure to slippery terrain under snow or rain can still trigger injuries indoors. Additionally, snowy and rainy days lead to behavioral changes due to thick clothes and protective gears. Our study was conducted using large-scale nationwide databases without analyzing clinical data of patients with emotional stress, alcohol use, and violence. Further, the effects of other natural events such as earthquakes leading to mass casualties were not considered.

In summary, we found that the incidence of FI is related to weather factors. Emergency medical personnel should understand that FIs occur frequently during days of low precipitation, high temperature and low winds linked with active outdoor activity in metropolitan areas. Additional weather factors have been shown to affect FI incidence in rural areas so that increased FI rates were noticed on days of low precipitation, high temperature, low winds and longer sunshine duration in rural areas. Moon phases are weakly linked to FI incidence rates. FIs increased only in rural areas during the full moon days compared with new moon days. FI severity is also affected by weather factors. In both urban and rural areas, the severity of FI significantly increased on cold and windy days.

## Supporting information

S1 Fig Areas to be studied were selected by dividing them into A) metropolitan areas (red color) including seven metropolitan cities with a population exceeding one million and B) rural areas (blue color) consisting of four provinces without containing metropolitan cities within its perimeter.
(PDF)

S2 Fig Distribution of daily fall injuries by weekday for metropolitan and rural areas. (PDF)
