# Supplementary material for: Effects of weather and moon phases on emergency medical use after fall injury: A population-based nationwide study
Source: PLoS One. 2021 Dec 31;16(12):e0261071. doi: 10.1371/journal.pone.0261071 (PMC8719656; doi:10.1371/journal.pone.0261071)
Supplement: S1 Fig — Areas to be studied were selected by dividing them into A) metropolitan areas (red color) including seven metropolitan cities with a population exceeding one million and B) rural areas (blue color) consisting of four provinces without containing metropolitan cities within its perimeter. (PDF) [file pone.0261071.s001.pdf]

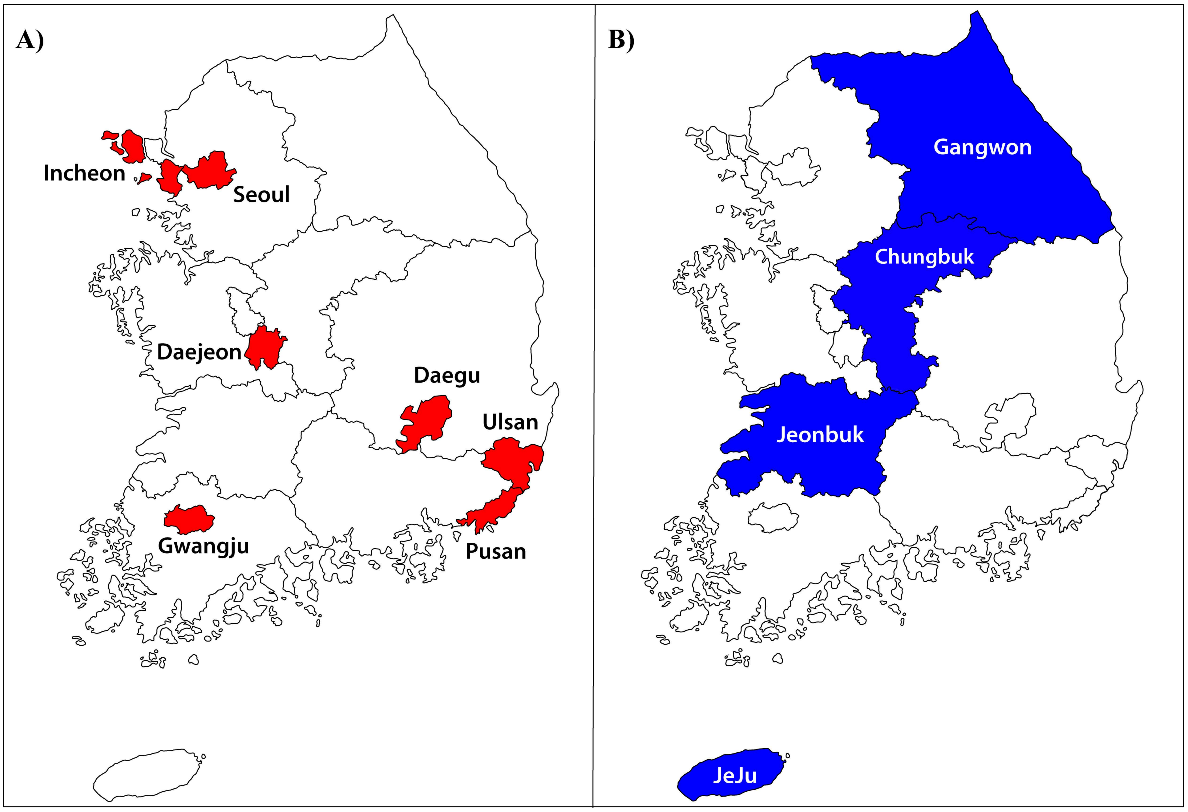

S1 Fig. Areas to be studied were selected by dividing them into A) metropolitan areas (red color) including seven metropolitan cities with a population exceeding one million and B) rural areas (blue color) consisting of four provinces without containing metropolitan cities within its perimeter. Reprinted from https://www.freepik.com/premium-vector/country-map-with-borders_8569611.htm under a CC BY license, with permission from Freepik company, 2021.
