# Supplementary material for: Effects of weather and moon phases on emergency medical use after fall injury: A population-based nationwide study
Source: PLoS One. 2021 Dec 31;16(12):e0261071. doi: 10.1371/journal.pone.0261071 (PMC8719656; doi:10.1371/journal.pone.0261071)
Supplement: S2 Fig — (PDF) [file pone.0261071.s002.pdf]

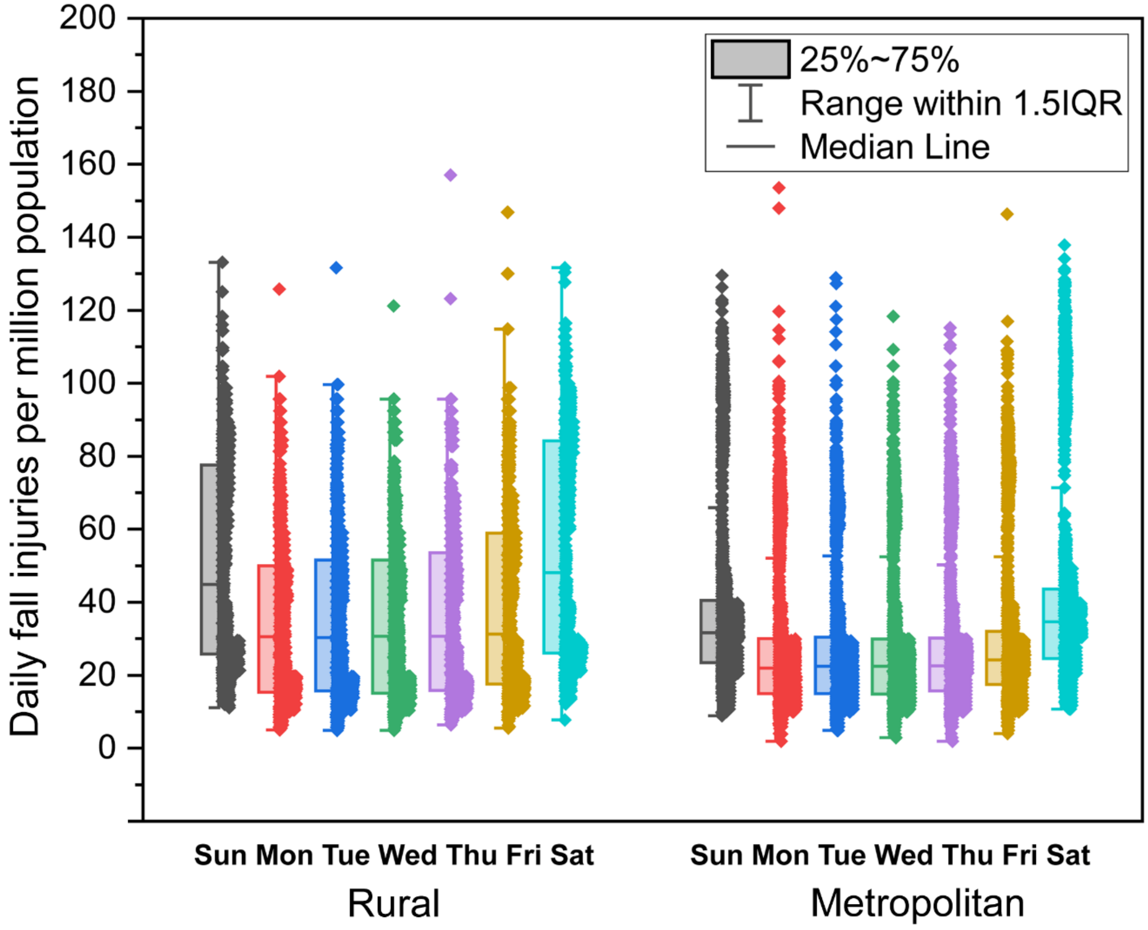

S2 Fig. Distribution of daily fall injuries by weekday for metropolitan and rural areas. The box is plotted on the left side and the scatterplot is illustrated on the right side. Numbers of fall injury patients per million population on Saturdays and Sundays were significantly higher than those on other days.
